# Iterative Schemes to Solve Low-Dimensional Calibration Equations in Parallel MR Image Reconstruction with GRAPPA

**DOI:** 10.1155/2017/3872783

**Published:** 2017-09-28

**Authors:** Omair Inam, Mahmood Qureshi, Shahzad A. Malik, Hammad Omer

**Affiliations:** Department of Electrical Engineering, COMSATS Institute of Information Technology, Islamabad, Pakistan

## Abstract

GRAPPA (Generalized Autocalibrating Partially Parallel Acquisition) is a widely used parallel MRI reconstruction technique. The processing of data from multichannel receiver coils may increase the storage and computational requirements of GRAPPA reconstruction. Random projection on GRAPPA (RP-GRAPPA) uses random projection (RP) method to overcome the computational overheads of solving large linear equations in the calibration phase of GRAPPA, saving reconstruction time. However, RP-GRAPPA compromises the reconstruction accuracy in case of large reductions in the dimensions of calibration equations. In this paper, we present the implementation of GRAPPA reconstruction method using potential iterative solvers to estimate the reconstruction coefficients from the randomly projected calibration equations. Experimental results show that the proposed methods withstand the reconstruction accuracy (visually and quantitatively) against large reductions in the dimension of linear equations, when compared with RP-GRAPPA reconstruction. Particularly, the proposed method using conjugate gradient for least squares (CGLS) demonstrates more savings in the computational time of GRAPPA, without significant loss in the reconstruction accuracy, when compared with RP-GRAPPA. It is also demonstrated that the proposed method using CGLS complements the channel compression method for reducing the computational complexities associated with higher channel count, thereby resulting in additional memory savings and speedup.

## 1. Introduction

Parallel imaging is an emerging technique to accelerate the MR data acquisition by undersampling the *k*-space data at each channel in multichannel coil arrays [1, 2]. The array coil with a large number of channels not only shortens scan session durations, but also provides improvements in signal-to-noise ratio (SNR) and better coverage of Field of View (FOV) [3–5]. In parallel imaging, undersampled data is acquired simultaneously by multiple channels and the image is reconstructed using parallel MRI (pMRI) techniques, for example, SENSE and GRAPPA [6, 7].

GRAPPA is a widely used *k-*space based pMRI technique [7]. GRAPPA interpolates the undersampled *k-*space data of multichannel receiver coils by estimating the unknown reconstruction coefficients from the fully acquired autocalibration signal (ACS). The reconstruction time of GRAPPA increases quadratically with the number of channels [8]. Large computation and memory requirements due to higher channel count also limit the efficiency and scalability of GRAPPA and other pMRI techniques on the computational platforms such as FPGAs and GPUs [9–12].

To mitigate the said problems, hardware and software based channel compression techniques are used [13–19]. These techniques linearly combine the input data from multichannel receiver coils to fewer channels, thereby significantly reducing the reconstruction time and the computer memory requirement. The channel reduction is implemented in hardware by constructing fewer Eigen coil arrays using an inline hardware combiner [19]. However this approach is often not optimal due to the additional hardware requirements. In contrast, software based channel reduction is more flexible and generates data with high SNR. The software channel compression techniques [13–18] reduce the number of physical channels to fewer virtual channels, by finding correlation between the physical channels with the help of principal component analysis (PCA).

Recently a different perspective has been presented in randomly projected GRAPPA (RP-GRAPPA) [20–22] to address the computational complexity of conventional GRAPPA reconstruction method [7]. Instead of using channel compression only, RP-GRAPPA advocates the use of random projection (RP) method to reduce the reconstruction time of conventional GRAPPA. During the calibration phase, RP-GRAPPA reduces the dimension of calibration equations and estimates the reconstruction coefficients by solving randomly projected calibration equations using pseudoinverse method. However, pseudoinverse method differs greatly from exact solution in case of large reductions in the calibration equations. Consequently RP-GRAPPA compromises the reconstruction accuracy [21, 22].

The purpose of this work is to enable the additional computational savings in the traditional GRAPPA reconstruction time by suppressing the reconstruction errors associated with randomly projected calibration equations as compared to RP-GRAPPA. Therefore, we introduced an alternative approach to solve the randomly projected calibration equations in the proposed methods, for rapid and robust GRAPPA reconstruction using potential iterative solvers (i.e., conjugate gradient for least squares (CGLS) and heuristic rule-based gradient descent (HGD) algorithms), instead of pseudoinverse method used in RP-GRAPPA. To the best of our knowledge, we present the first implementation of GRAPPA reconstruction method using CGLS and HGD to estimate the reconstruction coefficients from low-dimensional calibration equations. The proposed methods are referred to as RP-CGLS-GRAPPA and RP-HGD-GRAPPA using CGLS and HGD, respectively.

Several experiments on two-dimensional (2D) MR images using different number of physical channels (8, 12, and 30) are performed to evaluate the efficacy of the proposed methods (RP-CGLS-GRAPPA and RP-HGD-GRAPPA) in terms of reconstruction accuracy, computation time, and storage savings. Experimental results validate that the RP-CGLS-GRAPPA efficiently reconstructs the image, while maintaining the reconstruction accuracy against large reductions in the linear equations, when compared with RP-GRAPPA. The benefits of using the proposed methods are further explored by integrating the PCA based channel compression (CC) method [18] with RP-CGLS-GRAPPA and RP-HGD-GRAPPA, referred to as CC-RP-CGLS-GRAPPA and CC-RP-HGD-GRAPPA, respectively. The channel compression method is also integrated with RP-GRAPPA (referred to as CC-RP-GRAPPA) to compare the reconstruction results with CC-RP-CGLS-GRAPPA and CC-RP-HGD-GRAPPA. The results show that CC-RP-CGLS-GRAPPA and CC-RP-HGD-GRAPPA exhibit superior reconstruction quality even after large reductions in the calibration equations, when compared with CC-RP-GRAPPA. However, CC-RP-CGLS-GRAPPA is more effective in terms of reconstruction time and memory savings as compared to the reconstruction methods using only channel compression (i.e., CC-RP-GRAPPA, CC-CGLS-GRAPPA, and CC-HGD-GRAPPA).

## 2. Theory and Methods

### 2.1. Conventional GRAPPA

GRAPPA is a *k-*space based pMRI technique [7]. This method reconstructs the missing *k*-space data in each receiver coil using convolutional kernel estimated from the fully acquired autocalibration signal (ACS). ACS lines are sampled at Nyquist rate and collected from the center of the *k-*space in each receiver coil. GRAPPA reconstruction process has two discrete phases, that is, calibration and synthesis. During the calibration phase, all the training datasets are collected in source (**S**_*m*×*n*_) and target (**T**_*m*×*l*_) matrices, during kernel repetitions over ACS lines, and form a GRAPPA calibration equation as

(1) $$\begin{array}{c} T_{m\times l}=S_{m\times n}W_{n\times l}\;where\;\;m\gg n, \end{array}$$

where **W**_*n*×*l*_ represent the unknown coefficients (also called reconstruction coefficients or GRAPPA weight sets) for linear combination between the source (**S**) and target (**T**) data points. In calibration phase, conventional GRAPPA seeks least square fits to estimate the reconstruction coefficients (**W**):

(2) $$\begin{array}{c} \hat{w}=\underset{w}{\min}{\left\|\;Sw-t\;\right\|}^{2}. \end{array}$$

The problem in (1) is well overdetermined; therefore a direct method known as pseudoinverse can be used to estimate the best fit for (2):

(3) $$\begin{array}{c} W={\left(\;S^{H}S\;\right)}^{-1}\left(\;S^{H}T\;\right), \end{array}$$

where *H* denotes the conjugate transpose.

During the synthesis phase in GRAPPA, the estimated weight sets (**W**) in (3) are used to calculate the missing data points in the undersampled region of the *k-*space for each channel in the array coil.

It is demonstrated in [21] that the computational expense of complex-valued multiplication during the calibration phase dominates the total GRAPPA reconstruction time. Thus any computational savings during the calibration phase may contribute to the reduction of the GRAPPA reconstruction time.

### 2.2. Dimension Reduction via PCA

Principal component analysis (PCA) is a well-known and widely used linear dimension reduction technique which finds the linear projections of high dimensional data onto lower dimensional subspace, such that the variance of the data in the low-dimensional representation is maximized [23–28]. However it is important to note that the computation complexity of estimating PCA is *O*(*d*^2^*N*) + *O*(*d*^3^) [29], where *N* is the number of data points in *d* dimensions. The time complexity of PCA shows that this method may become infeasible for a problem with the large *d*.

In pMRI, PCA is widely used to compress the channels in a large array of receiver coils [15–18]. This technique significantly reduces the computer memory requirements and reconstruction time of pMRI techniques without considerable degradation in the quality of the reconstructed image. The computational overheads of PCA do not affect the performance of pMRI because the number of channels is not very large in general. Therefore, PCA is a suitable choice for channel compression in pMRI.

### 2.3. Dimension Reduction via Random Projection

Random projection (RP) method is a popular, computationally efficient, and sufficiently accurate dimensionality reduction technique used in many signal processing and machine learning applications [29–31]. RP is based on Johnson-Lindenstrauss lemma [32]. The lemma says that there exists a projection *f* that maps the set of *n* points in *m*-dimensional Euclidean space onto *k*-dimensional (*k* ≪ *m*) Euclidean subspace such that the distances between any two points are approximately preserved up to the factor (1 ± *e*). The main idea of RP is to map the original *d*-dimensional data **A** onto *k*-dimensional subspace (*k* ≪ *d*), using randomly generated *k* × *d* matrix **R**. The projection process can be expressed as

(4) $$\begin{array}{c} P_{k\times N}=R_{k\times d}\times A_{d\times N}. \end{array}$$

RP is computationally less expensive as compared to other dimension reduction methods such as PCA, as the projection process in (4) involves only one matrix-matrix multiplication of order *O*(*kdN*) [29]. However, the choice of projection matrix **R** in (4) is a key factor as it provides the mapping that satisfies Johnson-Lindenstrauss lemma and determines the complexity of the projection process. Achlioptas [32] proposed a simple probabilistic method to generate sparse projection matrix **R** that still satisfies Johnson lemma. A useful property of the Achlioptas distribution is the generation of a* sparse projection matrix ***R** with only (1/3)rd of the data to be processed, resulting in a threefold speedup in the projection process [32]. Later, Li et al. [33, 34] generalized the Achlioptas results and proposed a very-sparse projection matrix **R** with entries *r*_*ji*_ belonging to the following distribution:

(5) $$\begin{array}{c} r_{ji}=\sqrt{s}\left\{\begin{array}{cc} +1 & with\;\;prob.\;\;\frac{1}{2s}\\ 0 & with\;\;prob.\;\;1-\frac{1}{s}\\ -1 & with\;\;prob.\;\;\frac{1}{2s}. \end{array}\right. \end{array}$$

Li et al. demonstrated $\sqrt{d}$ fold speedup in the processing time [34], by using $s=\sqrt{d}$ in (5), where *s* = 3 and *s* = 1 in (5) are the cases for Achlioptas distribution.

### 2.4. Random Projection on GRAPPA (RP-GRAPPA)

Recently random projection on GRAPPA (RP-GRAPPA) [20–22] addressed the computational complexity of the calibration phase in conventional GRAPPA and used random projection (RP) method to reduce the dimensions of the problem in (1). It is demonstrated in [20–22] that the solution to the reduced set of equations during calibration phase of GRAPPA is approximately the same as the original one, provided that the value of *λ* is set appropriately.

(6) Rλn×mSm×nWn×l−Rλn×mTm×lF≈Sm×nWn×l−Tm×lF,

where *λ* defines a factor by which the order of source (**S**) and target matrices (**T**) in (1) are reduced. A small value of *λ* implies large reductions in the linear equations. RP-GRAPPA only focused on the pseudoinverse method to estimate the reconstruction coefficient (**W**), by solving a reduced set of linear equations as

(7) $$\begin{array}{c} W={\left(\;{\left(\;S_{red}\;\right)}^{H}\left(\;S_{red}\;\right)\;\right)}^{-1}\left(\;{\left(\;S_{red}\;\right)}^{H}\left(\;T_{red}\;\right)\;\right), \end{array}$$

where **S**_red_ = **R**_*λn*×*m*_**S**_*m*×*n*_ and **T**_red_ = **R**_*λn*×*m*_**T**_*m*×*l*_.

This approach significantly reduced the total reconstruction time of GRAPPA. However, RP-GRAPPA introduced large reconstruction errors if the value of *λ* is not set appropriately. It is recommended in [22] that the value of *λ* must be greater than 2.2 in order to avoid large reconstruction errors. In [22], the optimal value for *λ* to balance the tradeoff between reconstruction errors and reconstruction time is seen to be 3.

### 2.5. Heuristic Rule-Based Gradient Descent (HGD)

Gradient descent (GD) [35] is a classical optimization technique and can be used to solve (1) in the sense of least square. GD repeatedly invokes the update rule in (8) until the iterate sequence converges to the optimal solution:

(8) $$\begin{array}{c} x_{i+1}=x_{i}-\mu e_{i}, \end{array}$$

where

(9) ei=−SHb−Sxi,μ=2γ,b∈Ck×1=T1T2⋮Tm,x∈Cn×1=W1W2⋮Wn.

The step size (*μ*) may influence the speed of convergence. Different choices of *μ* lead to various gradient based algorithms [36, 37]. Heuristic rule-based Gradient Descent (HGD) algorithm [38] shown in Algorithm 1(a) dynamically updates *μ* and iteratively solves

(10) $$\begin{array}{c} x_{\left(i+1\right)}=x_{\left(i\right)}-\eta_{\left(i\right)}e_{\left(i\right)}, \end{array}$$

where *η*_(*i*)_ defines the learning rate [38]:

(11) $$\begin{array}{c} \eta =\frac{\mu }{\left\|e\right\|}. \end{array}$$

Starting with some initial value of *μ*_0_, HGD dynamically updates the value *μ* in (11) using two heuristic rules [38]. (i) HGD increases *μ* by a factor of 1.1 after experiencing four consecutive reductions in residual: ‖**b** − **Sx**_(**i** + 1)_‖. (ii) HGD decreases *μ* by a factor of 0.9 after observing two consecutive combinations of one increase and one reduction in residual. In HGD, the initial value of *μ* is not critical as long as it is not large enough.

### 2.6. Conjugate Gradient for Least Squares (CGLS)

Conjugate gradient (CG) algorithm belongs to the family of Krylov subspace iterative methods, for solving a symmetric positive definite (SPD) linear system and a linear least square problem [39, 40]. CG methods are characterized by their need of storing few vectors and better rate of convergence [40, 41]. Algorithm 1(b) shows that CGLS avoids explicit computation of matrix-matrix product (**S**^*H*^**S**) which causes bad performances in the case of ill-conditioned system. This method performs a sequential linear search along **S**^*H*^**S**-conjugate directions {**p**_0_, **p**_1_,…, **p**_**i**−1_} that spans the Krylov subspace:

(12) κiSHS,SHb=spanSHb,SHSSHb,…,SHSi−1SHb.

The* i*th iterate of CGLS solves the least square problem:

(13) xi=argminx∈κiSHS,SHb 12Sx−b22.

The update in **x**_**i**_ is given by **x**_**i**_ = **x**_**i**−1_ + *α*_*i*−1_**p**_**i**−1_, where *α*_*i*−1_ solves one-dimensional minimization problem:

(14) minα Sxi−1+αipi−1−b22.

The search direction vector **p**_**i**_ = **s**_**i**_ + *β*_*i*−1_**p**_**i**−1_ is updated using the residual error **s**_**i**_ = **S**^*H*^(**b** − **Sx**_**i**_) and the previous direction **p**_**i**−1_, where the parameter *β*_*i*−1_ is chosen so that **p**_**i**_ is **S**^*H*^**S**-conjugate to all the previous search directions; that is, **p**_**i**_^*H*^**S**^*H*^**Sp**_**j**_ = 0, 1 ≤ **j** ≤ *i* − 1.

### 2.7. Proposed Methods (RP-CGLS-GRAPPA and RP-HGD-GRAPPA)

RP-GRAPPA uses pseudoinverse method to solve reduced calibration equations in the calibration step as discussed in Section 2.4. If (**S**_red_) is ill-conditioned then the solution obtained by pseudoinverse method (see (7)) may differ greatly from the exact solution. It is due to the fact that *κ*((**S**_red_)^*H*^(**S**_red_)) = *κ*(**S**_red_)^2^ [42]. This implies that, in case of an ill-conditioned system, any perturbations in (**S**_red_) or rounding-off errors in the computed matrix ((**S**_red_)^*H*^(**S**_red_)) can introduce inversion errors which may result in poor estimation of the reconstruction coefficients (**W**) using (7). Consequently the quality of reconstruction suffers. Therefore we propose to use CGLS and HGD algorithms in RP-CGLS-GRAPPA and RP-HGD-GRAPPA, respectively, to iteratively solve the randomly projected calibration equations. The CGLS and HGD avoid the explicit computation of ((**S**_red_)^*H*^(**S**_red_)) matrix and works separately on (**S**_red_) and (**S**_red_)^*H*^ to generate a series of progressively improved GRAPPA weight sets in an iterative fashion. This approach has two important advantages: (i) it avoids the effect of large ((**S**_red_)^*H*^(**S**_red_)), which leads to the inversion errors in (7) and (ii) using (**S**_red_) in the context of multiplying by a vector it avoids much more expensive matrix-matrix multiplication in (7).

**Figure 1:**
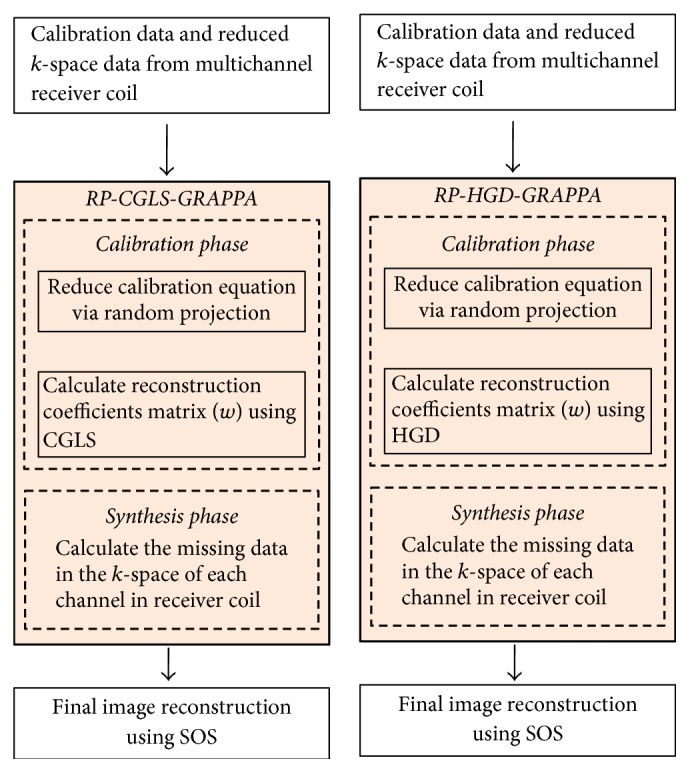
RP-CGLS-GRAPPA and RP-HGD-GRAPPA.

In the proposed methods, GRAPPA calibration is performed in two steps, shown in Figure 1.

**Step 1 .**

Dimension reduction via random projection method is applied in (1), to obtain a reduced set of linear equations (see (16)) (also referred to as randomly projected calibration equations) as follows:

(15) Sredk×n=Rk×mSm×n=Rλn×mSm×n,Tredk×l=Rk×mTm×l=Rλn×mTm×l,

where *k* ≪ *m*, *λ* = *k*/*n*, and *k* ≥ *n*.

Hence, (1) becomes

(16) $$\begin{array}{c} {T_{red}}_{k\times l}={S_{red}}_{k\times n}W_{n\times l}. \end{array}$$

The projection process in (15) uses very-sparse projection matrix **R** with *r*_*ji*_ entries belonging to (5), that is, Li et al.'s distribution where $s=\sqrt{m}$:

(17) $$\begin{array}{c} r_{ji}=\sqrt[{4}]{m}\left\{\begin{array}{cc} +1 & with\;\;prob.\;\;\frac{1}{2\sqrt{m}}\\ 0 & with\;\;prob.\;\;1-\frac{1}{\sqrt{m}}\\ -1 & with\;\;prob.\;\;\frac{1}{2\sqrt{m}}. \end{array}\right. \end{array}$$

**Step 2 .**

CGLS and HGD algorithms are used in the RP-CGLS-GRAPPA and RP-HGD-GRAPPA, respectively, to accurately estimate the reconstruction coefficients (**W**) by solving the randomly projected calibration equations shown in (16).

During the synthesis phase, missing data in the *k*-space of each channel is calculated by linearly combining the estimated reconstruction coefficients (**W**) and the acquired *k*-space data in source matrix (**S**). Once the fully sampled *k*-space has been estimated for all the receiver coils, a set of uncombined images for each coil is constructed using Fourier Transform. The composite image is then reconstructed using the sum-of-square reconstruction of the individual coil images.

The convergence rate of CGLS and HGD in the proposed methods depends upon the size and the condition number (*κ*) of the coefficient matrix (**S**) [41, 43]. Algorithm 1(a and b) shows that, during each iterate, complex-valued matrix-vector multiplications of order *O*(*mn*) and the storage of all previous searching directions and residual vectors may increase the computational complexity of HGD and CGL algorithms. It can be observed in Algorithm 1(a) that HGD requires two complex-valued matrix-vector multiplications (i.e., **Sx** and **S**^*H*^**r**) and working storage of two *n*-vectors (**e** and **x**) and two *m*-vectors (**r** and **q**), during each iterate. For CGLS (see Algorithm 1(b)) two complex-valued matrix-vector products (i.e.,** Sp** and **S**^*H*^**r**) and the working storage of two *n*-vectors (**x** and **p**), and two *m*-vectors (**r** and **q**), are needed during each iterate. To reduce the overall computation and storage complexity of HGD and CGLS, we apply random projection in the proposed methods (RP-CGLS-GRAPPA and RP-HGD-GRAPPA), to reduce the number of calibration equations (see (1)) and obtain a reduced linear system as shown in (16). With the reduced set of linear equations (*k* ≪ *m*), CGLS and HGD require *O*(*kn*) operations per iteration for complex-valued matrix-vector multiplications. Moreover, the working storage of CGLS and HGD is also reduced from two *m*-vectors to two *k*-vectors. The impact of random projection on the efficiency and reconstruction accuracy of the proposed methods is analyzed and discussed in Results and Discussion.

### 2.8. Integrating PCA Channel Compression with RP-CGLS-GRAPPA and RP-HGD-GRAPPA

**Figure 2:**
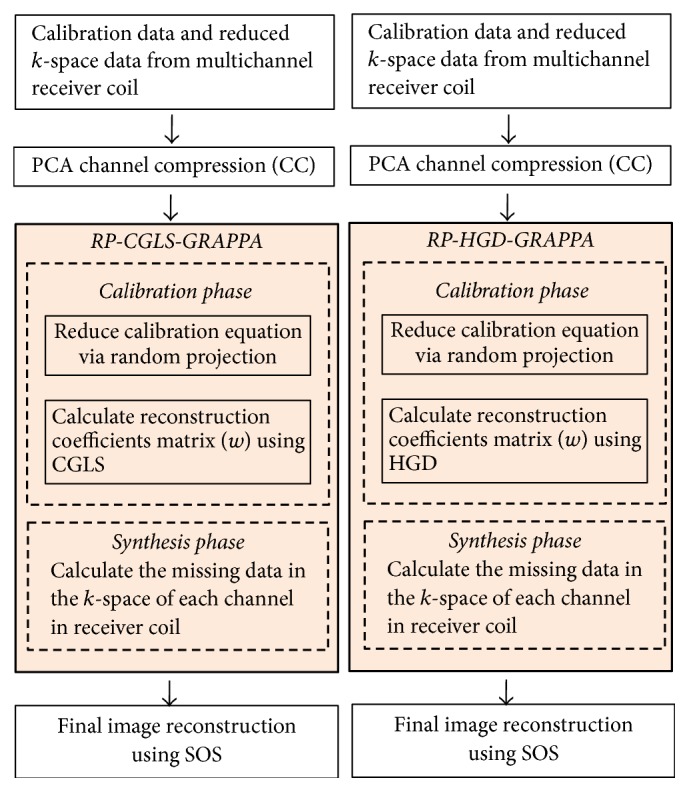
CC-RP-CGLS-GRAPPA and CC-RP-HGD-GRAPPA.

Principal component analysis (PCA) and random projection method (RP) are two popular linear techniques for dimension reduction with different properties and applications. PCA is suitable for channel compression in pMRI, as it removes the redundant information by decorrelating data from different channels, whereas RP involves the projection of high dimensional data onto the randomly selected subspace of a lower dimension, while preserving the pairwise distance in the original space. RP is a computationally efficient method as compared to PCA [29] and therefore a suitable choice to reduce the complexity of calibration step in GRAPPA. Therefore additional computational and memory savings can be achieved by integrating PCA channel compression (CC) [18] with RP-CGLS-GRAPPA and RP-HGD-GRAPPA as illustrated in Figure 2. For this purpose, CC is performed before GRAPPA reconstruction to reduce the number of channels to be processed and RP is applied during the calibration phase of the proposed methods to reduce the computational complexity associated with CGLS and HGD.

### 2.9. Data Acquisitions

The proposed methods are evaluated on three fully sampled in vivo datasets: (i) cardiac data acquired using 3.0 T Siemens Skyra scanner at Case Western Reserve University, Cleveland, OH, USA, with 30-channel receiver coils using cine based SSFP sequence, FOV = 300 mm^2^, TR/TE = 2/0.8 ms, slice thickness = 8 mm, flip angle = 50°, and matrix size = 512 × 252 with 11 frames. (ii) Human head data acquired from 3.0 T Siemens Skyra scanner with 12-channel head coil array, FOV = 230 mm^2^, TR/TE = 938.7/2 ms, slice thickness = 5 mm, flip angle = 58°, and matrix size = 448 × 224. (iii) Human head data acquired from 1.5 T GE scanner at St. Mary's Hospital London using 8-channel head coil with matrix size 256 × 256, FOV = 200 mm^2^, TR/TE = 500/10 ms, slice thickness = 3 mm, and flip angle = 50°. Healthy volunteers were examined after gaining informed written consent with the approval of Institutional Review Board for Human Studies at University Hospitals of Cleveland, Case Western Reserve University (CWRU), and St. Mary's Research Ethics Committee (REC).

### 2.10. Data Analysis

Retrospective undersampling for various acceleration factors *A*_*F*_ (3, 5, and 8) was performed on the fully sampled datasets. For the comparison purpose, reference images (gold standard) were obtained from the fully sampled data of all the receiver coils, using sum-of-square reconstruction. In this paper, projection process given in (15) uses a very-sparse matrix **R**, randomly generated from the distribution shown in (5) using $s=\sqrt{m}$. The difference images with reference and the *g*-factor maps for the combined GRAPPA images, beside Root Mean Square Error (RMSE), signal-to-noise ratio (SNR) and reconstruction time, are used to evaluate and compare the performance of the proposed methods with RP-GRAPPA, whereas the *g*-factor maps for the combined GRAPPA images are calculated based on Eq. [12] in [44]. The computational time and memory savings were measured in terms of CPU time required for GRAPPA reconstruction and the number of bytes required to store **S**_red_ and **T**_red_ matrices after dimension reduction. All the methods in this work were implemented in MATLAB (Mathworks, Natick, MA) and run on Intel(R) Core(TM) i5-3210M CPU @ 2.50 GHz, 2501 MHz, 2 Cores, and 4 logical processors with 4 GB Memory.

## 3. Results and Discussion

### 3.1. In Vivo Datasets Using 8- and 12-Channel Receiver Coils

We performed several experiments on in vivo datasets obtained using 8- and 12-channel receiver coils to validate the performance of the proposed methods (RP-CGLS-GRAPPA and RP-HGD-GRAPPA) in terms of reconstruction accuracy and computation time. For this, fully sampled *k*-space data was retrospectively undersampled in the phase encoding direction by a factor (*A*_*f*_) of 3 with 48 ACS lines. For 8- and 12-channel dataset, kernel sizes of 4 × 11 (4 along *d*_*y*_ and 11 along *d*_*x*_) and 4 × 7 (4 along *d*_*y*_ and 7 along *d*_*x*_) were used, respectively.

**Figure 3:**
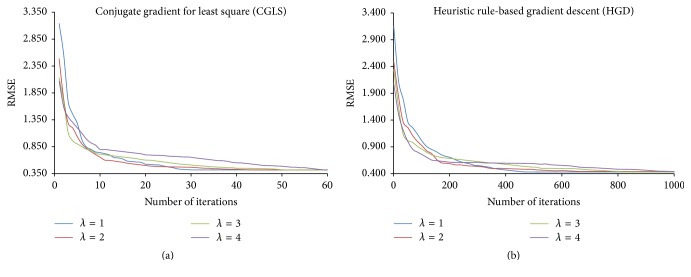
Convergence of the iterative methods for different values of *λ* to reconstruct the images using 8-channel dataset with 48 ACS lines, *A*_*f*_ = 3, and kernel size 4 × 11: (a) CGLS algorithm; (b) HGD algorithm.

**Figure 4:**
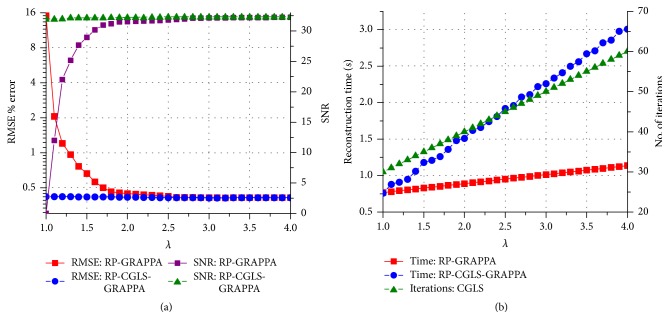
(Eight-channel dataset with 48 ACS lines, *A*_*f*_ = 3, and kernel size 4 × 11) (a) RMSE and SNR of RP-GRAPPA and RP-CGLS-GRAPPA, versus *λ*; (b) reconstruction time of RP-GRAPPA and RP-CGLS-GRAPPA, and the convergence behavior of CGLS, in terms of number of iterations, versus *λ*.

**Figure 5:**
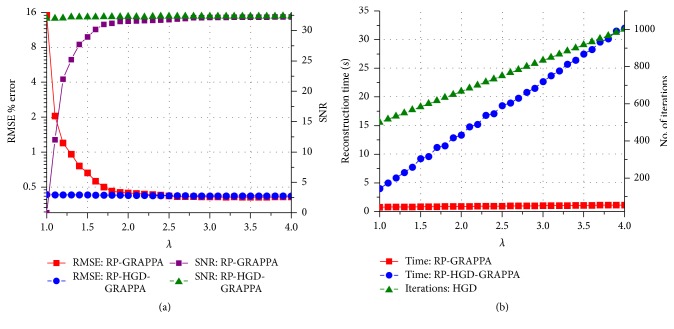
(Eight-channel dataset with 48 ACS lines, *A*_*f*_ = 3, and kernel size 4 × 11) (a) RMSE and SNR of RP-GRAPPA and RP-HGD-GRAPPA, versus *λ*; (b) reconstruction time of RP-GRAPPA and RP-HGD-GRAPPA and the convergence behavior of the HGD, in terms of number of iterations, versus *λ*.

**Figure 6:**
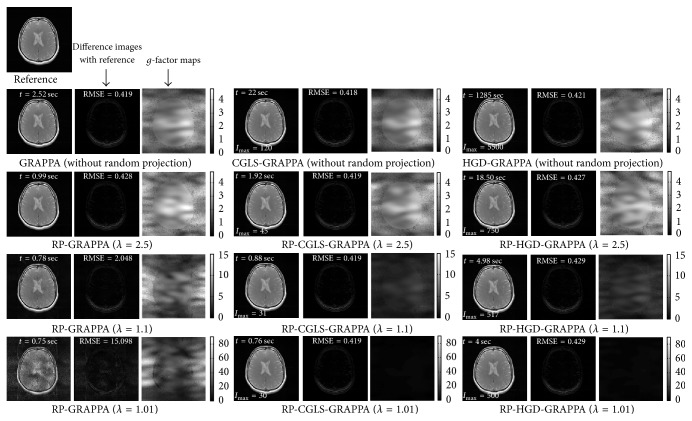
Axial brain images reconstructed from 8-channel dataset with acceleration factor (*A*_*f*_) = 3, kernel size 4 × 11, and 48 ACS lines, using conventional GRAPPA, RP-GRAPPA, CGLS-GRAPPA, RP-CGLS-GRAPPA, HGD-GRAPPA, and RP-HGD-GRAPPA. The difference images and *g*-maps are shown along the corresponding reconstructed images. In the first row, all the reconstructions are performed without using random projection. Second, third, and fourth rows show the reconstruction results with *λ* = 2.5, 1.1, and 1.01, respectively.

**Figure 7:**
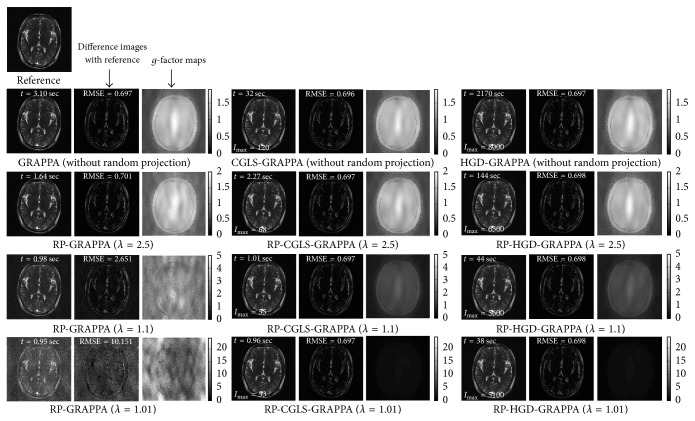
Brain images reconstructed from 12-channel dataset with acceleration factor (*A*_*f*_) = 3, kernel size 4 × 7, and 48 ACS lines, using conventional GRAPPA, RP-GRAPPA, CGLS-GRAPPA, RP-CGLS-GRAPPA, HGD-GRAPPA, and RP-HGD-GRAPPA. The difference images and *g*-maps are shown along the corresponding reconstructed images.

We evaluated the proposed methods and RP-GRAPPA using 8-channel dataset to investigate the effect of reducing *λ* in the range between 4 and 1, on the computational time, reconstruction accuracy, and convergence behavior of CGLS and HGD algorithms. For this purpose, the convergence of CGLS and HGD algorithms is analyzed against the reduction in the calibration equations by varying the reduction parameter *λ* (in the range between 4 and 1), where the small value of *λ* implies large reductions in the calibration equations. For a particular value of *λ* (in the range between 4 and 1) the convergence behaviors of CGLS and HGD algorithms are found experimentally by performing the image reconstructions using different number of iterations (*I*_max_) as shown in Figures 3(a) and 3(b) where the convergence of CGLS and HGD algorithm is illustrated only for *λ* = 1, 2, 3, and 4. It can be observed in Figure 3(a) that for *λ* = 1 the proposed method using CGLS algorithm reconstructs the image with comparable image quality in terms of RMSE, using minimum of 30 iterations. In this case (*λ* = 1) the quality of image reconstruction remains the same even if the number of iterations is further increased. Therefore, for each value of *λ* between 4 and 1 the minimum number of iterations required by the CGLS and HGD algorithms to achieve a comparable reconstruction quality in terms of RMSE is estimated experimentally and plotted in Figures 4(b) and 5(b) along the reconstruction time of proposed methods. Due to the random nature of the RP-CGLS-GRAPPA, RP-HGD-GRAPPA, and RP-GRAPPA, all the curves in Figures 4 and 5 are obtained by averaging the reconstruction results (RMSE, SNR, and reconstruction time) from 50 experiments. In Figures 4(a) and 5(a), RP-GRAPPA shows rapid increase in RMSE and almost exponential decay in the values of SNR of the reconstructed images, for *λ* < 2.2. However, in the case of RP-CGLS-GRAPPA (Figure 4(a)) and RP-HGD-GRAPPA (Figure 5(a)), the RMSE and SNR values of the reconstructed images remain almost steady for *λ* ranging between 4 and 1. In Figures 4(b) and 5(b), the reconstruction time of the proposed methods and the number of iterations (CGLS and HGD) are plotted against *λ*. The results show that the reconstruction time of RP-CGLS-GRAPPA and RP-HGD-GRAPPA decreases with the number of iterations due to the reductions in the calibration equation.  Figure 4(b) exhibits that the reconstruction time of RP-CGLS-GRAPPA is reduced from 3 to 0.76 sec as the number of iterations (*I*_max_) for CGLS is decreased from 60 to 30. Moreover, in Figures 4(b) and 5(b), a linear trend in the reconstruction time of RP-CGLS-GRAPPA and RP-HGD-GRAPPA validates the large reductions in the computational complexity of CGLS and HGD. It is due to the fact that the random projection method (RP) significantly reduces the computational overheads of iterative algorithms (CGLS, HGD), by minimizing the computational complexity of matrix-vector multiplication (*O*(*kn*)) during each iterate. However, RP-CGLS-GRAPPA performs better in terms of reconstruction time as compared to RP-HGD-GRAPPA due to a better convergence rate of CGLS. In Figures 6 and 7, the reconstruction results using 8- and 12-channel datasets are illustrated for visual and quantitative comparisons of the proposed methods and RP-GRAPPA with and without random projections. The results demonstrate that the when the value of *λ* is set at 1.01, RP-GRAPPA tempered the reconstruction accuracy by increasing the RMSE of conventional GRAPPA to 3503% (Figure 6) and 1356% (Figure 7). However, both RP-CGLS-GRAPPA and RP-HGD-GRAPPA methods showed superior reconstruction quality (visually and quantitatively), when compared with RP-GRAPPA for *λ* = 1.1 and 1.01. The reconstruction results in Figures 4, 6, and 7 clearly show that the RP-CGLS-GRAPPA achieved the maximum reduction in the reconstruction time of GRAPPA without compromising the quality of image reconstruction at *λ* = 1.01; that is, RP-CGLS-GRAPPA speeds up the GRAPPA reconstruction time by factor of 2.52/0.76 = 3.3x at *λ* = 1.01 (see Figure 6). However, Figures 4 and 5 suggest that, in the case of RP-GRAPPA, the optimal value of *λ* to balance the tradeoff between the RMSE, SNR, and computational time is 2.5. Therefore the maximum achievable speedup by RP-GRAPPA with comparable image quality is reported (see Figure 6) at *λ* = 2.5, that is, 2.52/0.99 = 2.5x. Similarly, it can be observed in Figure 7 that for *λ* = 1.01 RP-CGLS-GRAPPA demonstrates high quality image reconstruction with speedup of 3.10/0.96 = 3.2x, whereas RP-GRAPPA achieved the maximum speedup of 3.10/1.64 = 1.8x at *λ* = 2.5 with comparable image quality. Furthermore, the reconstruction results in Figures 4, 6, and 7 demonstrate that RP-GRAPPA never approaches the same reconstruction quality with the same maximum possible speedup of RP-CGLS-GRAPPA (*λ* = 1.01). For example, the results in Figures 6 and 7 demonstrate that both RP-GRAPPA and RP-CGLS-GRAPPA have almost the same computation time at *λ* = 1.01, for example, 0.75 sec and 0.76 sec, respectively, in Figure 6 and 0.95 sec and 0.96 sec, respectively, in Figure 7; however, the RMSE value of RP-GRAPPA at *λ* = 1.01 increases to an unacceptable level as shown in Figures 6 and 7, resulting in poor reconstruction quality as compared to RP-CGLS-GRAPPA. Therefore it is evident from the results shown in Figures 4, 6, and 7 that the RP-CGLS-GRAPPA is a suitable choice to improve the efficiency of GRAPPA reconstruction with high quality image reconstruction.

**Figure 8:**
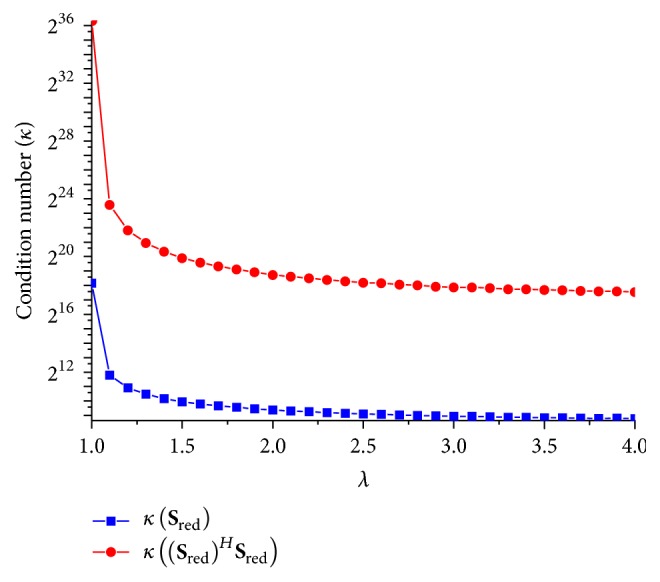
Condition number of (**S**_red_) and ((**S**_red_)^*H*^(**S**_red_)) versus *λ*, for 8-channel dataset with ACS = 48, *A*_*F*_ = 3, and kernel size 4 × 11.

In the proposed methods, the iterative techniques (i.e., CGLS and HGD) are preferred over direct method (i.e., pseudoinverse method) to avoid the inversion errors arising (using (7)) in the case of an ill-conditioned system (i.e., if the condition number of computed matrix ((**S**_red_)^*H*^(**S**_red_)) in (7) is large). For this, we investigated the effect of reducing the calibration equation on the condition number of the computed matrix ((**S**_red_)^*H*^(**S**_red_)).  Figure 8 shows that the condition number of the computed matrix ((**S**_red_)^*H*^(**S**_red_)) used in (7) shows quadratic growth as *λ* decreases from 2.5 to 1. Due to the quadratic rise in the condition number of ((**S**_red_)^*H*^(**S**_red_)), the inversion errors in (7) (pseudoinverse method) become dominant which results in poor estimation of the reconstruction coefficients. Consequently the quality of RP-GRAPPA reconstruction suffers. This can be validated by comparing the results of RP-GRAPPA in Figures 4 and 5 with the results plotted in Figure 8, which show that for *λ* < 2.5 the reconstruction errors in RP-GRAPPA increased exponentially with the quadratic rise in the condition number of computed matrix ((**S**_red_)^*H*^(**S**_red_)). On the other hand, CGLS and HGD algorithms do not require the explicit computation of ((**S**_red_)^*H*^(**S**_red_)) matrix thus avoiding the effect of large condition number of ((**S**_red_)^*H*^(**S**_red_)) on the reconstruction quality of the proposed methods particularly for *λ* < 2.5. Therefore, CC-RP-CGLS-GRAPPA demonstrates additional speedup as compared to RP-GRAPPA, by enabling robust reconstruction against the variation of *λ* between 2.5 and 1.

### 3.2. In Vivo Datasets Using 30-Channel Receiver Coils

**Figure 9:**
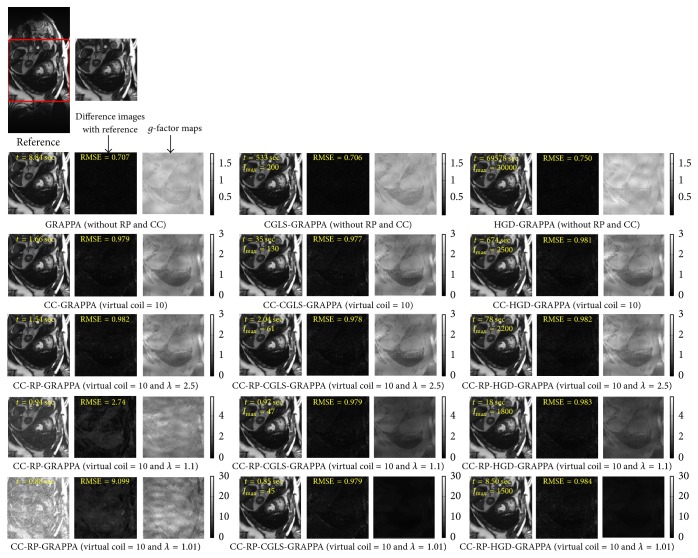
Reconstruction results of 30-channel cardiac dataset using GRAPPA, CC-GRAPPA, CC-RP-GRAPPA, CGLS-GRAPPA, CC-CGLS-GRAPPA, CC-RP-CGLS-GRAPPA, HGD-GRAPPA, CC-HGD-GRAPPA, and CC-RP-HGD-GRAPPA with acceleration factor (*A*_*F*_) = 5, kernel size 4 × 5, and 48 ACS lines. The 2nd row shows the reconstruction results of the methods using only coil compression. PCA based coil compression was used to reduce 30 channels to 10 virtual channels. The difference images and *g*-maps are shown along the corresponding reconstructed images. 3rd, 4th, and 5th row show the reconstruction results of the integrated methods using *λ* = 2.5, 1.1, and 1.01.

**Figure 10:**
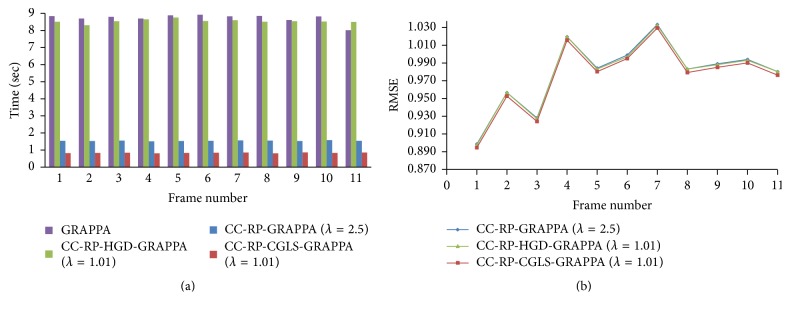
The reconstruction time and quality of reconstructed images (in terms of RMSE) are plotted for all the frames in 30-channel cardiac dataset to compare the performances of the integrated methods using acceleration factor (*A*_*F*_) = 5, kernel size 4 × 5, and 48 ACS lines. (a) Reconstruction time of each frame using GRAPPA, CC-RP-HGD-GRAPP, CC-RP-GRAPPA, and CC-RP-CGLS-GRAPPA: (b) RMSE of each reconstructed frame using GRAPPA, CC-RP-HGD-GRAPP, CC-RP-GRAPPA, and CC-RP-CGLS-GRAPPA.

**Figure 11:**
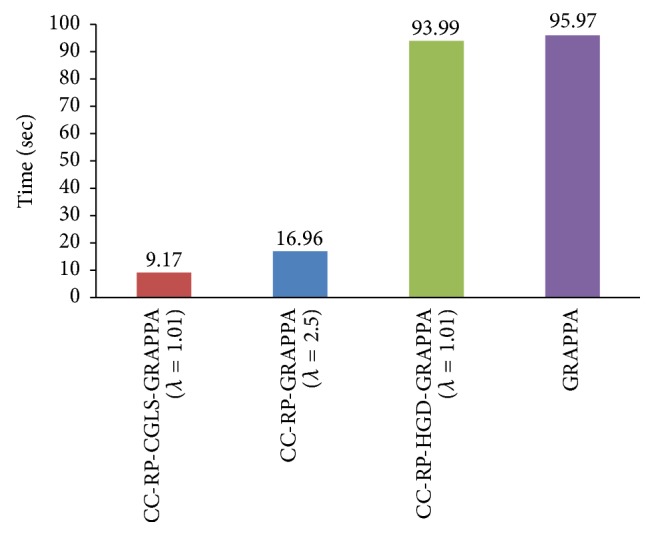
Total time to reconstruct all the frames of 30-channel cardiac dataset by GRAPPA, CC-RP-HGD-GRAPP, CC-RP-GRAPPA, and CC-RP-CGLS-GRAPPA using acceleration factor (*A*_*F*_) = 5, kernel size 4 × 5, and 48 ACS lines.

**Figure 12:**
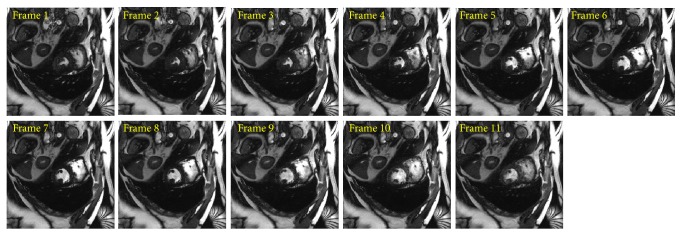
Reconstructed images of all 11 frames in 30-channel cardiac dataset, by CC-RP-CGLS-GRAPPA (at *λ* = 1.01) using acceleration factor (*A*_*F*_) = 5, kernel size 4 × 5, and 48 ACS lines. PCA based coil compression was used to reduce 30 channels to 10 virtual channels.

PCA based channel compression method is a well-known choice to reduce the computational complexity of pMRI techniques due to higher channel count. Additional computational and memory savings can be achieved by integrating PCA based channel compression (CC) with the proposed methods. For this purpose one fully sampled frame (i.e., frame number 11) of cardiac dataset was retrospectively undersampled with *A*_*f*_ = 5 and 8 to perform the reconstructions and compare the performance of the proposed methods (i.e., CC-RP-CGLS-GRAPPA and CC-RP-HGD-GRAPPA) and RP-GRAPPA integrated with channel compression method (CC-RP-GRAPPA), in terms of reconstruction time, accuracy, and memory savings. In Figure 9, the reconstruction results with *A*_*f*_ = 5 demonstrate that the CC-RP-CGLS-GRAPPA achieved maximum speedup of 8.84/0.85 = 10.4x in the reconstruction time at *λ* = 1.01, whereas, in the case of CC-RP-GRAPPA, the maximum achievable speedup without compromising the quality if image reconstruction is seen at *λ* = 2.5, that is, 8.84/1.54 = 5.70x. It can be observed from Figure 9 that CC-RP-GRAPPA never approaches the same reconstruction quality with the same maximum possible speedup of CC-RP-CGLS-GRAPPA. The results in Figure 9 demonstrate that, at *λ* = 1.01, both CC-RP-GRAPPA and CC-RP-CGLS-GRAPPA have almost the same computation time, that is, 0.88 sec and 0.85 sec, respectively, whereas, at *λ* = 1.01 the RMSE of CC-RP-GRAPPA is increased to an unacceptable level, resulting in poor reconstruction quality as compared to CC-RP-CGLS-GRAPPA. Furthermore, all 11 frames of 32 channel cardiac dataset are reconstructed for *A*_*f*_ = 5 using CC-RP-CGLS-GRAPPA (*λ* = 1.01) and CC-RP-GRAPPA (*λ* = 2.5). To compare the performance of the proposed techniques with CC-RP- GRAPPA, the reconstruction time and RMSE of all frames are plotted in Figures 10(a) and 10(b). The reconstruction results in Figure 10(a) shows that the CC-RP-CGLS-GRAPPA performs consistently better in terms of reconstruction time than other techniques. Moreover, the total time to reconstruct all frames of the 32-channel cardiac dataset is plotted in Figure 11. The results show that the CC-RP-GRAPPA consumes 16.96 sec to reconstruct all the frames with comparable quality, whereas CC-RP-CGLS-GRAPPA requires 9.17 sec for the reconstruction of all the frames of 32-channel cardiac dataset. The reconstruction results of CC-RP-CGLS-GRAPPA (*λ* = 1.01) are also shown in Figure 12.

**Figure 13:**
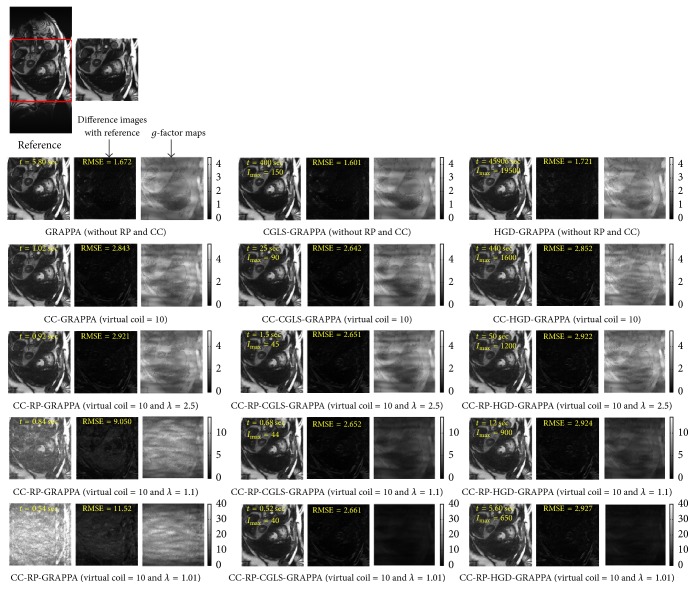
Reconstruction results of 30-channel cardiac dataset using GRAPPA, CC-GRAPPA, CC-RP-GRAPPA, CGLS-GRAPPA, CC-CGLS-GRAPPA, CC-RP-CGLS-GRAPPA, HGD-GRAPPA, CC-HGD-GRAPPA, and CC-RP-HGD-GRAPPA with acceleration factor (*A*_*F*_) = 8, kernel size 4 × 5, and 48 ACS lines. The 2nd row shows the reconstruction results of the methods using only coil compression. PCA based coil compression was used to reduce 30 channels to 10 virtual channels. The difference images and *g*-maps are shown along the corresponding reconstructed images. 3rd, 4th, and 5th row show the reconstruction results of the integrated methods using *λ* = 2.5, 1.1, and 1.01.

**Figure 14:**
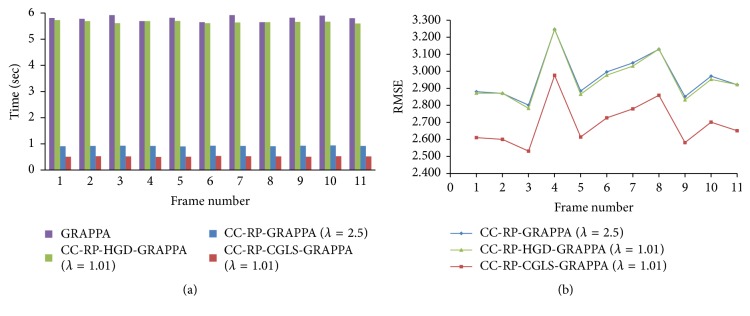
The reconstruction time and quality of reconstructed images (in terms of RMSE) are plotted for all the frames in 30-channel cardiac dataset to compare the performances of the integrated methods using acceleration factor (*A*_*F*_) = 8, kernel size 4 × 5, and 48 ACS lines. (a) Reconstruction time of each frame using GRAPPA, CC-RP-HGD-GRAPP, CC-RP-GRAPPA, and CC-RP-CGLS-GRAPPA: (b) RMSE of each reconstructed frame using GRAPPA, CC-RP-HGD-GRAPP, CC-RP-GRAPPA, and CC-RP-CGLS-GRAPPA.

**Figure 15:**
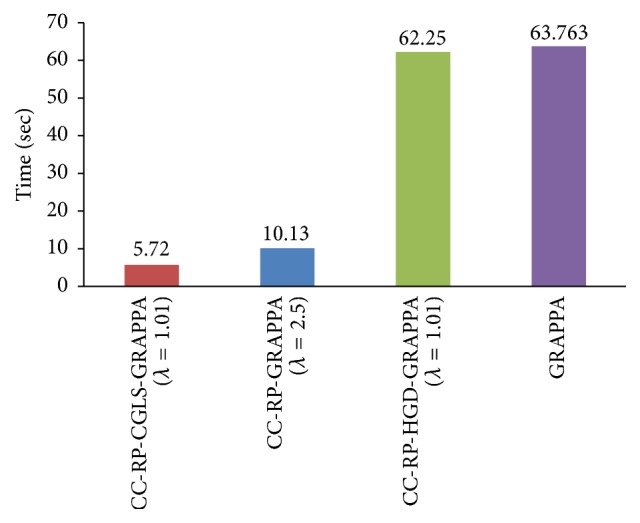
Total time to reconstruct all the frames of 30-channel cardiac dataset by GRAPPA, CC-RP-HGD-GRAPP, CC-RP-GRAPPA, and CC-RP-CGLS-GRAPPA using acceleration factor (*A*_*F*_) = 8, kernel size 4 × 5, and 48 ACS.

**Figure 16:**
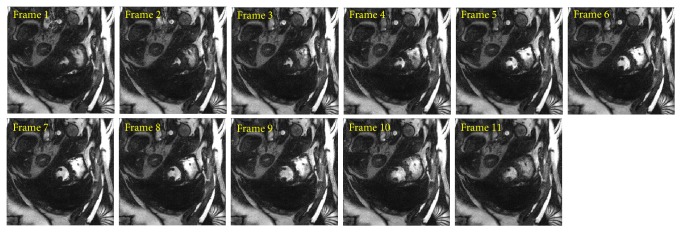
Reconstructed images of the all the 11 frames in 30-channel cardiac dataset, by CC-RP-CGLS-GRAPPA (at *λ* = 1.01) using acceleration factor (*A*_*F*_) = 8, kernel size 4 × 5, and 48 ACS. PCA based coil compression was used to reduce 30 channels to 10 virtual channels.

For a higher acceleration factor, that is, *A*_*f*_ = 8, the CC-RP-CGLS-GRAPPA (*λ* = 1.01) (in Figure 13) demonstrates 5.80/0.52 = 11.1x speedup with high quality image reconstruction (Figure 14), whereas CC-RP-GRAPP (*λ* = 2.5) achieved a maximum of 5.80/0.92 = 6.3x speedup with comparable image quality.  Figure 15 shows that the CC-RP-GRAPPA (*λ* = 2.5) requires 10.13 sec to reconstruct all the frames with comparable image quality (Figure 16), whereas CC-RP-CGLS-GRAPPA (*λ* = 1.01) reconstructs all the frames of 32-channel cardiac dataset in only 5.72 sec. Therefore, CC-RP-CGLS-GRAPPA is a suitable choice to improve the efficiency of GRAPPA reconstruction with high quality image reconstruction.

In addition to the traditional GRAPPA which is only used for Cartesian trajectories, the CGLS and HGD algorithms with random projections are also applicable to any parallel MRI reconstruction technique that involves solving a large, overdetermined linear equation. For example, it can be applied to most of the GRAPPA extensions for Cartesian and non-Cartesian trajectories [45–48]. However, if the calibration phase in the pMRI techniques does not dominate the total reconstruction time as in the case of SPIRiT [49], then the computational savings are limited.

### 3.3. Memory Savings due to Reduction in Calibration Equations

**Figure 17:**
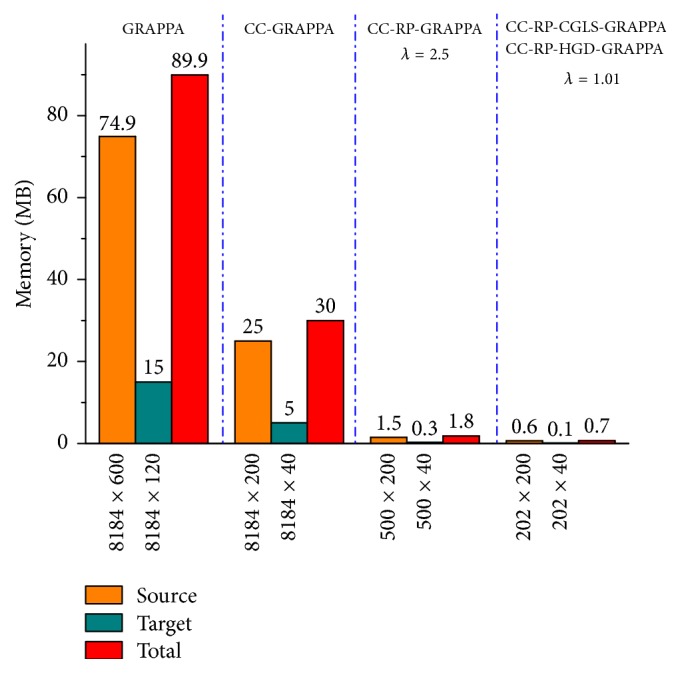
Memory savings with respect to the size of source and target matrices in the conventional GRAPPA, CC-GRAPPA, CC-RP-GRAPPA, CC-RP-CGLS-GRAPPA, and CC-RP-HGD-GRAPPA using 30-channel cardiac dataset with 48 ACS lines, *A*_*f*_ = 5, and kernel size 4 × 5.

Due to the amplification of reconstruction errors for *λ* < 2.2, RP-GRAPPA puts a limit on the reduction in the dimension of source (**S**_red_) and target (**T**_red_) matrices (in (7)). However, the proposed methods (RP-CGLS-GRAPPA and RP-HGD-GRAPPA) allow more reductions in the dimensions of (**S**_red_) and (**T**_red_), without significant loss in the reconstruction accuracy, when *λ* is used between 1 and 2.2. Therefore, the memory cost is estimated with respect to the dimension of matrices (**S**_red_) and (**T**_red_) to demonstrate the benefits of integrating channel compression with random projection for GRAPPA reconstruction. It is demonstrated in Figure 17 that when only channel compression was used in conventional GRAPPA (CC-GRAPPA), the order of the source and target matrices is reduced from 8184 × 600 (74.9 MB) to 8184 × 200 (25 MB) and 8184 × 120 (15 MB) to 8184 × 40 (5 MB), respectively. Hence, the total memory requirement to store both the matrices is reduced from 89.9 MB to 30 MB. In the case of RP-GRAPPA the optimal value to balance the tradeoff between the reconstruction error and computation time is found at *λ* = 2.5; therefore the total storage cost in the case of CC-RP-GRAPPA is estimated at *λ* = 2.5, that is, 1.8 MB. In the case of CC-RP-CGLS-GRAPPA and CC-RP-HGD-GRAPPA, the total memory cost is reduced from 89.9 MB to 0.7 MB only.

The low memory requirement during calibration and inherent parallelism in CGLS are useful characteristics that can be considered for future work to further improve the efficiency and scalability of the proposed methods on devices like FPGAs and GPUs.

## 4. Conclusions

In this work, we proposed two methods (i.e., RP-CGLS-GRAPPA and RP-HGD-GRAPPA) using iterative solvers (i.e., CGLS and HGD algorithms) for the robust reconstruction against the variation of *λ* (parameter used for dimension reduction in random projection) between 2.5 and 1 and to achieve an additional speedup in the GRAPPA reconstruction time as compared to RP-GRAPPA. Experimental results demonstrated that the RP-CGLS-GRAPPA is a suitable choice to improve the efficiency of GRAPPA reconstruction with high quality image reconstruction. Furthermore, it was shown that the RP-CGLS-GRAPPA complemented the channel compression for providing additional computational and memory savings without compromising the reconstruction accuracy.

## Figures and Tables

**Algorithm 1:**
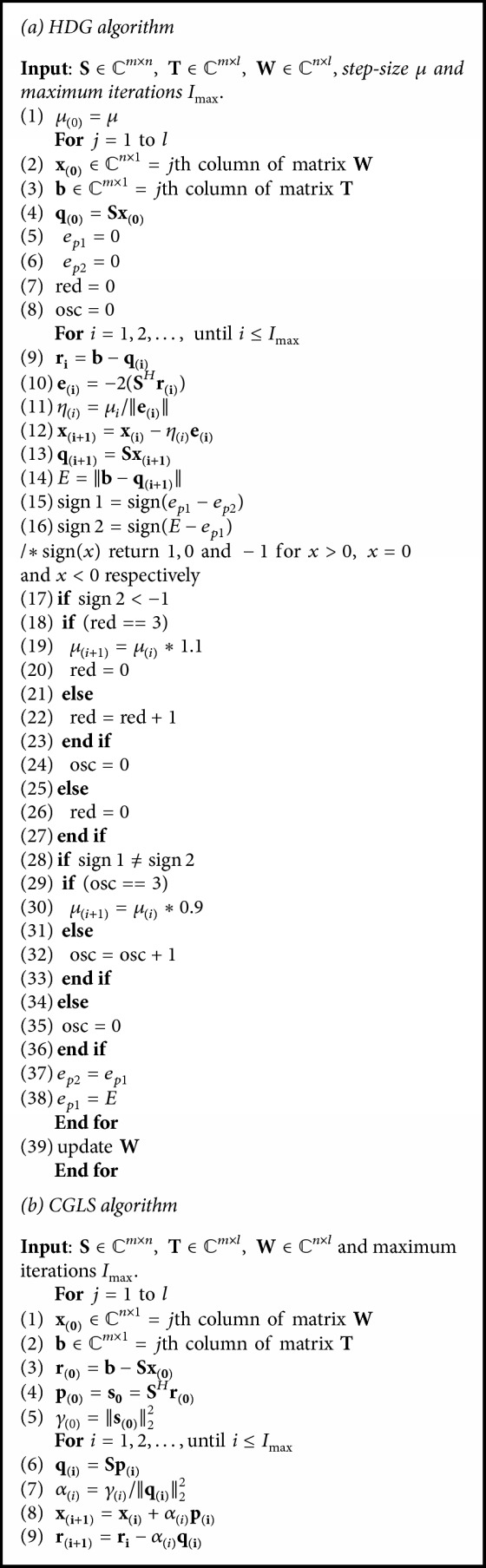
(a) Heuristic rule-based gradient descent (HGD); (b) conjugate gradient for least squares (CGLS).
